# Environmental Detection of Pathogenic *Leptospira* DNA in Agricultural Ecosystems from a Mediterranean-Climate Region of Central Chile

**DOI:** 10.3390/pathogens15070661

**Published:** 2026-06-23

**Authors:** M. Fernanda San Martin, Nicol Quiroga, Arnau Casanovas-Massana, Carezza Botto-Mahan, Antonella Bacigalupo, Pedro E. Cattan, Patricio Arroyo, Juan Contardo, Rodrigo Salgado, Esteban Yefi-Quinteros, Juana P. Correa

**Affiliations:** 1 Facultad de Ciencias Veterinarias y Pecuarias, Universidad de Chile, Santiago 8820808, Chile; fersanmartinsartori@gmail.com (M.F.S.M.); pcattan@uchile.cl (P.E.C.); patricioarroyo@gmail.com (P.A.); rodrigo.salgadomoya@gmail.com (R.S.); estebanyefi@gmail.com (E.Y.-Q.); 2 Facultad de Ciencias, Universidad de Chile, Santiago P.O. Box 653, Chile; nicol.quiroga.h@gmail.com (N.Q.); cbotto@uchile.cl (C.B.-M.); 3 Research Ring in Pest Insects and Climate Change (PIC2), Santiago 8380453, Chile; abacigalupo@udla.cl; 4 Department of Epidemiology of Microbial Diseases, Yale School of Public Health, New Haven, CT 06510, USA; acasanovasmassana@gmail.com; 5 Facultad de Medicina Veterinaria y Agronomía, Universidad de Las Américas, Santiago 7500975, Chile; 6 Laboratório de Biologia Estrutural, Instituto Butantan, Butantã, São Paulo CEP 05503-900, Brazil; j.laclote.proppg@proppg.butantan.gov.br; 7 Facultad de Medicina Veterinaria, Universidad San Sebastián, Concepción 4080871, Chile

**Keywords:** *Leptospira*, leptospirosis, environmental detection, qPCR, soil, water, agricultural ecosystems, Mediterranean climate, irrigation, Chile

## Abstract

Although pathogenic *Leptospira* DNA has been detected in water and soil from different climatic regions, information from Mediterranean-climate agricultural systems remains limited. This study characterized the environmental detection of pathogenic *Leptospira* DNA in water and soil samples from irrigated agroecosystems of central Chile, evaluating spatial and seasonal variation and associations with selected physicochemical variables. A total of 605 samples were collected from eight agricultural sites during spring 2019, summer 2020, and winter 2021. Samples were analyzed by real-time PCR targeting *lipL32.* Overall, 29.1% of samples were PCR-positive, and pathogenic *Leptospira* DNA was detected in all sites and seasons. Soil samples showed higher positivity than water samples (34.5% vs. 21.4%), and positivity was higher in summer (41.7%) than in spring (22.7%) or winter (19.3%). Water temperature and turbidity were the only physicochemical variables that differed between positive and negative samples, whereas the binomial generalized linear mixed model (GLMM) showed that season and sample type were associated with PCR positivity after accounting for site-level clustering. These results show that pathogenic *Leptospira* DNA can be widely detected in irrigated agricultural systems from a Mediterranean-climate region, suggesting that soil, seasonality, irrigation practices, and other site-level characteristics should be considered in future studies on the environmental ecology of pathogenic *Leptospira*.

## 1. Introduction

Pathogenic species of the genus *Leptospira* are widely distributed and can infect several vertebrate species, including humans [1,2]. Infection can occur directly through contact with biological fluids from infected animals, usually urine, or, more frequently, through indirect contact with contaminated environments, particularly soil and water, where the bacteria may persist [1,3,4]. In this sense, contaminated environmental matrices may represent an interface among animal reservoirs, wildlife, domestic animals, and humans [4,5,6]. 

Previous studies have associated the environmental detection of pathogenic *Leptospira* with variables such as temperature, pH, humidity, and turbidity [3,5,6,7,8,9,10]. However, these associations are not always consistent among ecosystems or climatic regions [5]. This suggests that the presence of the bacteria in a given environment may not only be explained by the physicochemical conditions of soil or water but also by ecological processes operating at different spatial and temporal scales.

Although environmental pathogenic *Leptospira* has been reported in tropical, subtropical, and temperate regions [3,5,10,11,12], information from Mediterranean-climate regions remains limited, particularly in agricultural ecosystems. Mediterranean-type climates occur in different regions of the world, including the Mediterranean Basin, California, central Chile, the Cape region of South Africa, and southern Australia, and are characterized by marked seasonality, with mild, wet winters followed by a prolonged dry season and hot summers [13,14]. This seasonal pattern differentiates Mediterranean regions from many other temperate climates, where water availability may be more evenly distributed throughout the year [13,14]. Because pathogenic *Leptospira* is sensitive to desiccation and depends on suitable environmental moisture conditions [5,6], the prolonged summer drought typical of Mediterranean regions may restrict its environmental persistence. However, in these regions, economic activities such as agriculture often depend on supplemental water irrigation. Irrigation may generate microhabitats that partially buffer the restrictive conditions associated with dry Mediterranean climates and favor the local environmental detection of the bacteria [13,15]. These agroecosystems may also support a wide diversity of potential hosts attracted by the availability of resources such as food, water, or shelter [16,17,18,19,20,21].

In central Chile, a Mediterranean-climate region, *Leptospira* infection has been reported in humans and in domestic and wild animals [16,22,23,24]. However, information on the environmental detection of pathogenic *Leptospira* in this region remains scarce, despite the presence of potential hosts and irrigated agricultural systems [16,22]. In this context, this study provides a descriptive and exploratory assessment of the environmental detection of pathogenic *Leptospira* in water and soil samples from agricultural ecosystems of central Chile. The study aimed to evaluate spatial and temporal variation in bacterial detection and to explore its potential associations with selected physicochemical characteristics and site-level variables.

## 2. Materials and Methods

### 2.1. Study Area

**Figure 1:**
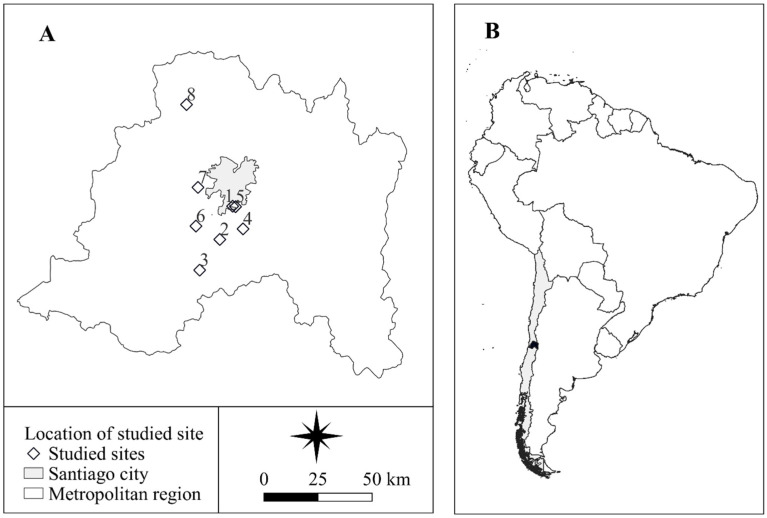
Location of the agricultural sites sampled for environmental detection of pathogenic *Leptospira* in central Chile. (**A**) Sampling sites within the Metropolitan Region, with Santiago city shown as a reference. (**B**) Location of the Metropolitan Region within South America.

The study area was located in the Metropolitan Region of Chile (Figure 1), a temperate region with Mediterranean influence, characterized by cold and rainy winters and scarce precipitation during the rest of the year. Summer is the season with the highest mean temperatures, whereas spring and fall have intermediate temperatures [25]. The region includes Santiago, the capital city of Chile, and is composed of urban areas as well as agricultural and livestock systems within a central valley surrounded by mountain ranges, including the Andes [26]. Given this climatic regime, most crops are maintained through artificial irrigation [27,28].

### 2.2. Sample Collection

The eight agricultural sites sampled in this study were separated by inter-site distances ranging from 1.4 km to 76.8 km. All sites had irrigation systems and showed variable presence of domestic animals. Sites were visited during the austral spring 2019 (September–November), summer 2020 (January), and winter 2021 (August). Georeferenced water and soil samples were collected at each site using a Global Positioning System device (UTM 19H, ±4 m accuracy) to determine the presence of *Leptospira* DNA, as well as to characterize selected physicochemical variables. Detailed information on each site is provided in Table 1, according to the available field information.

Detailed point-level microhabitat characteristics were not systematically recorded for all sampling points. Therefore, the description of sampling points was limited to the general criteria used during fieldwork: water samples were collected from available water sources such as irrigation ditches, canals, puddles, and water storage ponds, whereas soil samples were collected from moist soil within each agricultural site. Sampling points were generally selected to be approximately 20 m apart whenever field conditions allowed, although this distance varied according to accessibility and the availability of suitable environmental matrices. The same sites were surveyed across the three seasons; however, the specific sampling points within each site did not always correspond to the same coordinates among campaigns. This was due to changes in field conditions, including the presence of temporary water bodies, changes in accessibility associated with farm management practices, and the availability of suitable environmental matrices for sampling. Therefore, seasonal comparisons were interpreted at the study-site and sample-type level, rather than as strict repeated measurements of identical georeferenced points. 

Water samples (40–45 mL) were collected directly from different water sources using sterile nuclease-free tubes. Soil samples (approximately 300 g) were collected from moist sites and stored in sterile sealed bags. Both water and soil samples were collected during the morning and transported to the laboratory on the same day in a cooler with ice packs.

### 2.3. Physicochemical Characterization of Environmental Samples

The physicochemical variables evaluated in water samples were pH, temperature (°C), and electrical conductivity (µS/cm), which were measured directly using a multiparameter meter (Yalitech, model AM 006, Santiago, Chile) according to the manufacturer’s instructions. Turbidity was measured in nephelometric turbidity units (NTU) using a turbidimeter (Yalitech, model TM007 Yalitector, Santiago, Chile).

For soil samples, pH and moisture content were measured based on the methodology proposed by Sadzawka et al. [29]. Soil pH was estimated using a multiparameter meter in a soil–water suspension prepared with 15 g of soil and 37.5 mL of water. Soil moisture content was estimated as the percentage difference between the weight of a 10–20 g soil aliquot before and after oven-drying at 105 °C for six hours (Biobase, model BOVV70F, Jinan, Shandong, China).

### 2.4. Molecular Detection of Pathogenic Leptospira DNA

All collected samples were concentrated before DNA extraction. For this procedure, 40 mL of water or 5 g of soil were processed following the methodologies proposed by Schneider et al. [30] and Casanovas-Massana et al. [31], with the modification that centrifugation was performed at a maximum speed of 10,000 rpm for 20 min. The resulting pellets were stored in nuclease-free microcentrifuge tubes at −20 °C until DNA extraction. DNA was extracted using a commercial kit (DNeasy PowerSoil; Qiagen, Hilden, Germany), eluted in 100 µL of elution buffer, and stored at −20 °C until molecular detection of *Leptospira*.

To detect *Leptospira* DNA, each sample was analyzed in duplicate by real-time PCR using the LipL32-45F and LipL32-286R primers, which amplify a 242 bp fragment of the *lipL32* gene [32]. The reaction mix contained 1× HOT FIREPol® EvaGreen® qPCR Mix Plus (Solis BioDyne, Tartu, Estonia), 0.4 µM of each primer, 2 µL of extracted DNA, 0.2 µg/µL bovine serum albumin (Invitrogen, Thermo Fisher Scientific, Waltham, MA, USA) [33], and nuclease-free water (Invitrogen, Thermo Fisher Scientific, Waltham, MA, USA) to a final volume of 20 µL. The amplification protocol consisted of an initial denaturation step at 95 °C for 12 min, followed by 45 cycles of 95 °C for 10 s, 46 °C for 15 s, and 72 °C for 30 s, with a final extension step at 72 °C for 5 min. A standard melting curve analysis was performed at the end of the reaction. In each PCR run, DNA from *L. interrogans* (strain Ballico of serovar australis, kindly provided by Public Health Institute of Chile) was included as a positive control, and nuclease-free water was included as a no-template control. A sample was considered positive when at least one duplicate reaction amplified with the expected melting profile [34].

To assess PCR inhibition, a real-time PCR assay targeting a plasmid added before DNA extraction was used as an internal amplification control (IAC) [35]. The IAC reaction was performed using the same qPCR chemistry, with primers specific for the internal plasmid control. The thermal profile consisted of an initial denaturation step at 95 °C for 12 min, followed by 40 cycles of 95 °C for 20 s and 60 °C for 30 s, ending with a standard melting curve analysis. All PCR assays were performed on the QuantStudio® 3 Real-Time PCR System (Thermo Fisher Scientific, Waltham, MA, USA).

### 2.5. Data Analysis

The proportion of sites with at least one *Leptospira*-positive sample was calculated, as well as the proportion of PCR-positive samples among positive sites. The proportion of positive samples was also calculated by sample type and season.

Fisher–Freeman–Halton exact test, also referred to as the extended Fisher’s exact test, was used to explore the association between PCR positivity and categorical variables, including sample type (soil or water), sampling season (winter, spring, or summer), and irrigation system. For categorical variables with more than two levels, significant Fisher’s exact tests were followed by pairwise Fisher’s exact tests using the pairwiseNominalIndependence function from the rcompanion R package. *p*-values were adjusted using the Benjamini–Hochberg false discovery rate procedure. Because the irrigation system was defined at the site level, its association with *Leptospira* positivity was evaluated as an exploratory ecological analysis rather than as an individual sample-level predictor. 

Differences in physicochemical variables between PCR-positive and PCR-negative samples were evaluated separately for water and soil samples, according to the variables measured in each matrix. Because physicochemical variables did not follow a normal distribution, comparisons between PCR-positive and PCR-negative samples were performed using the Wilcoxon rank-sum test, also known as the Mann–Whitney U test [36]. In addition, as an exploratory complementary analysis, seasonal differences in physicochemical variables were evaluated using the Kruskal–Wallis test. When significant differences were detected, post hoc pairwise comparisons were performed using Dunn’s test with Benjamini–Hochberg false discovery rate correction. 

Finally, to account for the non-independence of samples collected within the same agricultural site, a generalized linear mixed model (GLMM) with binomial error distribution and logit link function was used to evaluate whether sampling season and sample type were associated with PCR positivity. Sampling site was included as a random intercept, whereas sampling season and sample type were included as fixed effects. Winter and water were used as reference categories. Model overdispersion was evaluated using Pearson residuals, and singular fit was assessed to verify the adequacy of the random-effect structure. Samples were classified as PCR-positive according to the predefined qPCR positivity criteria. As a sensitivity analysis, the main mixed-effects model was repeated using a stricter positivity definition, considering as positive only those samples with two positive PCR replicates.

Because the irrigation type was defined at the site, this variable was evaluated as an exploratory site-level predictor. To assess whether irrigation type improved model fit, an additional GLMM including irrigation type as a fixed effect was compared with the main model using Akaike information criterion (AIC), Bayesian information criterion (BIC), and a likelihood ratio test. 

Statistical analyses were performed in R version 4.5.3 [37], with a significance level of α = 0.05.

## 3. Results

A total of 605 environmental samples were analyzed, including 248 water samples and 357 soil samples. The number of samples collected per site ranged from 30 to 145, and the number of samples collected per season ranged from 88 to 299 (Table A1). 

The IAC amplified in all samples, indicating that PCR inhibition was not detected. Overall, 29.1% of samples were PCR-positive for pathogenic *Leptospira*. Pathogenic *Leptospira* DNA was detected in at least one sample from all sampled sites, with site-level positivity ranging from 14.2% to 43.3% when both sample types were combined (Table A1). 

As an exploratory site-level analysis, samples from sites with surface irrigation showed higher positivity than samples from sites with drip irrigation (33.1% vs. 22.9%; Fisher’s exact test, *p* = 0.008). Because each irrigation system was represented by four sites (Table 1), and irrigation type was defined at the site level rather than the sample level, this result was interpreted as an ecological site-level association.

**Figure 2:**
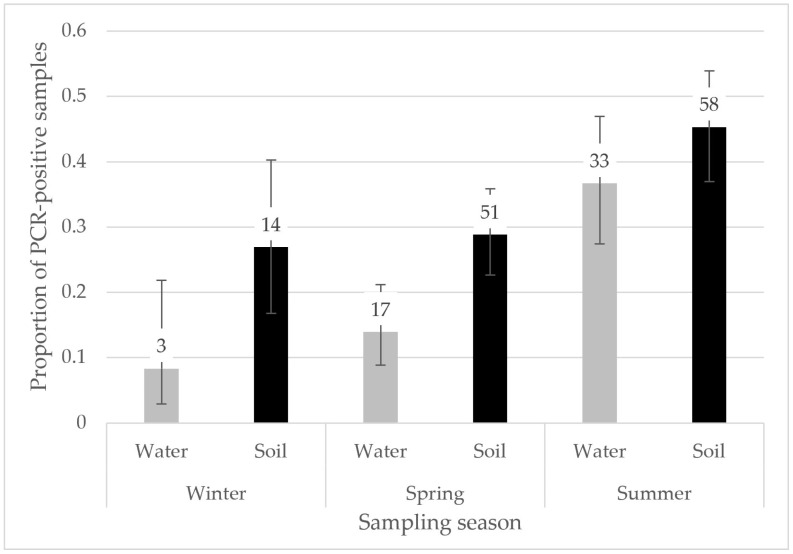
Proportion of PCR-positive samples for pathogenic *Leptospira* by sampling season and environmental matrix in agricultural ecosystems of central Chile. Bars represent the proportion of PCR-positive samples in water and soil collected during winter, spring, and summer (data from all sites combined). Error bars represent 95% binomial confidence intervals calculated using the Wilson method. Numbers above bars indicate the number of PCR-positive samples in each category.

Soil samples showed a higher frequency of positivity than water samples (34.5% vs. 21.4%; Fisher’s exact test, *p* < 0.001). Pathogenic *Leptospira* DNA was detected in all three sampled seasons, with positivity ranging from 19.3% to 41.7% when both sample types were combined (Figure 2, Table A1). PCR positivity was associated with sampling season (Fisher’s exact test, *p* < 0.001), with a higher frequency of positive samples in summer (41.7%) than in spring or winter (22.7% and 19.3%, respectively).

Among the physicochemical variables evaluated, temperature and turbidity differed between PCR-positive and PCR-negative water samples. Both temperature and turbidity were higher in PCR-positive than in PCR-negative water samples (Wilcoxon rank-sum test; W = 3410.5, *p* < 0.001, for temperature; W = 3378, *p* = 0.013, for turbidity) (Table 2). Both variables also differed among sampling seasons, with higher values during summer and lower values during winter (Table 2). 

The binomial GLMM, which accounts for site-level clustering by including sampling site as a random intercept, showed that both season and sample type were associated with the probability of *Leptospira* detection (Table 3). The model did not show evidence of singular fit, and no overdispersion was detected based on Pearson residuals (ratio = 0.98, *p* = 0.64). The standard deviation of the random intercept for the sampling site was 0.42.

Compared with winter, samples collected during summer had three times higher odds of being PCR-positive (OR = 3.25, *p* < 0.001), whereas spring did not differ significantly from winter (OR = 1.37, *p* = 0.31). Soil samples had approximately twice the odds of positivity compared with water samples, after adjusting for sampling season (OR = 2.13, *p* < 0.001). The interaction between season and sample type was evaluated, but it did not improve model fit; therefore, the additive model was retained. 

When irrigation type was added to the mixed-effects model, the direction of the association was consistent with higher PCR positivity in sites with surface irrigation; however, this effect was not statistically significant after accounting for site-level clustering (OR = 1.58, *p* = 0.14). In addition, adding the irrigation type did not significantly improve model fit compared with the model including only season, sample type, and sampling site as a random intercept (likelihood ratio test: χ^2^ = 1.79, df = 1, *p* = 0.18; AIC: 690.59 vs. 690.38; BIC: 717.02 vs. 712.41). Therefore, the simpler model was retained as the final model.

The sensitivity analysis using a stricter positivity criterion yielded consistent results with the main model. Summer and soil samples remained significantly associated with higher PCR positivity. Specifically, samples collected during summer showed higher odds of PCR positivity than those collected during winter (OR = 2.67, *p* = 0.028), and soil samples showed higher odds of positivity than water samples (OR = 2.55, *p* = 0.003). In contrast, spring did not differ significantly from winter.

## 4. Discussion

The main finding of this study was the widespread detection of pathogenic *Leptospira* DNA across heterogeneous agricultural ecosystems in the Mediterranean-climate region of central Chile. The *lipL32* fragment was detected at all sampled sites, in both soil and water, across all evaluated seasons. This molecular detection most likely reflects environmental contamination by *Leptospira* cells shed by infected hosts; however, because culture, PMA-PCR, RNA-based assays, or other viability-oriented methods were not performed, qPCR results cannot determine whether those cells were viable at the time of sampling. Therefore, these results should not be interpreted as direct evidence of viable bacteria or infective risk [5,11].

Because the sites studied differed in irrigation systems, crop types, and recorded animal presence, these results suggest that pathogenic *Leptospira* DNA can be detected across different agricultural contexts within this Mediterranean-climate region. This is particularly relevant because irrigated agricultural systems may create local environmental conditions that differ from those expected under Mediterranean summer drought. Nevertheless, given the exploratory and descriptive nature of this study, the observed patterns should not be interpreted as evidence of causal associations among the environmental factors evaluated. The odds ratios obtained from the GLMM should therefore be interpreted as tentative, model-based summaries of association within the sampled environmental matrices, rather than as causal or population-level estimates.

The patterns observed in the descriptive analyses were supported by GLMM. When the site was included as a random effect to account for the non-independence of samples collected within the same agricultural site, both sample type and season remained associated with PCR positivity. Specifically, soil samples and samples collected during summer had higher odds of being PCR-positive. These results suggest that the observed differences by matrix and season were not solely attributable to site-level clustering, although they should be interpreted within the limits of the field-sampling design.

The higher positivity observed in soil samples is consistent with previous studies reporting more frequent detection of *Leptospira* in soils, sediments, or shore substrates than in open water [5,9,38,39,40]. Moist soils may retain *Leptospira* cells or DNA more effectively than water, whereas water samples may be more influenced by dilution, flow, and transient contamination. Therefore, the higher soil positivity found in this study suggests that irrigated agricultural soils may represent relevant matrices for environmental detection of pathogenic *Leptospira* DNA in Mediterranean-climate agroecosystems. Soil positivity obtained in this study was comparable to values reported in tropical settings, such as Taiwan and Brazil [30,41], and higher than values described in some arid, subtropical, or temperate regions [42,43].

Although soil samples showed higher positivity than water samples, the detection of pathogenic *Leptospira* DNA in water should not be overlooked. In humid south-central Chile, pathogenic *Leptospira* DNA has been detected in peri-domestic surface waters from rural farms, rural villages, and urban settlements, showing that water sources can be contaminated in different Chilean landscape contexts [11]. In the present study, the 21.4% positivity observed in water samples indicates that these matrices are also relevant for environmental surveillance in irrigated Mediterranean-climate agroecosystems. Water positivity in this study was within the range reported in studies from different regions [11,12,31,40,42,43,44,45,46,47], with values closer to those found in livestock or agricultural settings, or in areas used by potential hosts, where environmental contamination through urine could be more likely [11,12,42,46]. 

Season was also associated with PCR positivity, with higher detection in summer. Seasonal differences in environmental *Leptospira* DNA detection have been reported elsewhere, for example, in tropical Brazil, where higher positivity in water samples was associated with the rainy season, probably because rainfall may disrupt soil biofilms and transport bacteria through water [31]. In contrast, the highest positivity in the present study occurred during the warmest and driest season. This result may seem counterintuitive in a Mediterranean-type climate, where summer rainfall is very limited. However, irrigation may maintain humid conditions in soil and water sources during the warmest months, potentially combining higher temperatures with local moisture availability (Table 2) [15,48]. This combination may favor environmental contamination, retention, or detection of pathogenic *Leptospira* DNA in agricultural matrices. In contrast, lower winter temperatures may have contributed to lower detection of *Leptospira* DNA, despite greater seasonal water availability. This interpretation should be considered tentative because irrigation was not experimentally manipulated and may be associated with other site-level agricultural characteristics.

The measured physicochemical variables had limited ability to differentiate between PCR-positive and PCR-negative samples. Water temperature and turbidity were higher in PCR-positive samples, but both variables were also closely related to sampling season, indicating that this association should be interpreted within the seasonal context of the study. In contrast, water conductivity, soil moisture, and pH did not differ between PCR-positive and PCR-negative samples, despite their seasonal variation. Other potentially important variables, including organic matter, dissolved oxygen, soil characteristics, vegetation cover, rainfall history, farm management practices, potential host communities, surrounding landscape structure, or topography, were not systematically measured [5,9,11,30,49,50,51]. 

Irrigation remains an important ecological feature to consider, but its role should be interpreted cautiously. In the exploratory analyses, PCR positivity varied according to irrigation type; however, irrigation was defined at the site level and not at the sample level, and the model including irrigation type did not improve the fit after accounting for site-level clustering. Therefore, irrigation should be interpreted as a plausible ecological factor that may influence local moisture, water movement, and connectivity among wet microhabitats [15,52], rather than as an independent driver demonstrated by this study. Other site characteristics, such as crop type, farm management, presence of animals, microbial community structure, or unmeasured microenvironmental differences, may also have contributed to the observed pattern [5,10,16,53].

Potential animal hosts are also relevant for interpreting environmental detection. Agricultural landscapes can support domestic animals, synanthropic rodents, and wildlife, which may contribute to environmental contamination through urine when infected [4,5,54,55]. In this study, animal presence was recorded descriptively at some sites, and previous studies have also reported rodents, including species with previous molecular evidence of pathogenic *Leptospira* infection, in some of the sites included in this work (Table 1) [16,17]. Thus, environmental detection most likely reflects contamination by *Leptospira* cells shed by infected hosts. However, host abundance, infection status, urinary shedding, and animal movement were not evaluated during environmental sampling. Consequently, the relative contribution of animal hosts to the PCR positivity observed in soil and water cannot be determined and should be considered a hypothesis for future studies. In addition, the summer peak observed in environmental samples does not fully coincide with previous rodent studies in south-central or central Chile, where higher seropositivity or infection frequencies were reported in winter or spring and lower values in summer or fall [17,56,57]. These contrasting patterns suggest that environmental PCR positivity may not directly mirror host infection frequency, because it can also depend on host abundance, habitat use, urinary shedding, animal movement, and local environmental conditions [20,54,55,58,59,60]. 

Agricultural systems such as those studied here may provide a useful framework for understanding the environmental ecology of pathogenic *Leptospira* because they combine several factors that can influence contamination from infected hosts, retention, and redistribution of pathogenic *Leptospira* cells or DNA in soil and water [5,9,49,52]. Compared with less-managed habitats, irrigated agroecosystems may include artificial water inputs, irrigation channels, ponds, animal drinking sites, cultivated soils, organic inputs, and variable presence of domestic animals, rodents, and wildlife [11,49,52,54,55]. These characteristics may maintain humid microhabitats during periods of low rainfall, promote contact between potential hosts and environmental matrices, and favor the redistribution of contaminated soil or organic material toward water sources through irrigation, runoff, or flooding events [9,49,52,55,59]. Therefore, the detection patterns observed in this study may reflect the combined influence of agricultural water management, soil moisture, host activity, and site-level management practices. However, these mechanisms were not directly evaluated here and should be considered as hypotheses for future studies rather than as causal explanations demonstrated by the present data. 

Overall, this study provides previously unavailable data for agroecosystems of Mediterranean-climate central Chile, showing widespread environmental detection of pathogenic *Leptospira* DNA across sites, sample types, and seasons. The main tentative patterns identified were higher PCR positivity in soil samples and during summer, even after accounting for site-level clustering. However, the observational nature of this work, the limited number of sites, and the absence of detailed host, microbial community, landscape, and farm-management data limit the interpretation of the mechanisms underlying these patterns. Future studies should explicitly integrate environmental sampling with host sampling, irrigation practices, soil and water characteristics, landscape structure, and seasonal management variables to better understand the biotic and abiotic factors associated with pathogenic *Leptospira* detection in Mediterranean-climate agroecosystems.

## 5. Conclusions

Pathogenic *Leptospira* DNA was widely detected in environmental samples from agricultural ecosystems in Mediterranean-climate central Chile, providing molecular evidence of environmental contamination despite the seasonal drought that characterizes this climate type.

The main tentative patterns identified were higher positivity in soil samples and during summer. Together with the limited explanatory capacity of the measured physicochemical variables, these findings suggest that environmental detection of pathogenic *Leptospira* DNA in these agroecosystems may be influenced by ecological and management-related processes operating at different scales, including irrigation practices, host activity, and other site-level characteristics.

Future studies should integrate environmental sampling with host data, irrigation practices, soil and water characteristics, landscape variables, and farm-management information to better understand the conditions associated with pathogenic *Leptospira* detection in Mediterranean-climate agricultural systems and to support preventive strategies.

## Figures and Tables

**Table 1:** Characteristics of the agricultural sites sampled in central Chile for the environmental detection of pathogenic *Leptospira*. Information includes agricultural use, water sources present at each site, predominant irrigation system, and animal species observed during fieldwork or previously reported in published studies conducted at the same sites [16,17]. Historical animal records from previous studies are indicated with an asterisk.

Site	Agricultural Use	Water Sources Present	Irrigation System	Animals Observed/Reported *
1	Mixed vegetable	Irrigation ditches, canals, and water storage ponds	Surface irrigation	Livestock/rodents *
2	Alfalfa–vineyard	Irrigation ditches, canals, and water storage ponds	Drip irrigation	Not recorded
3	Plum and walnut trees	Canal	Drip irrigation	Rodents *
4	Apple trees	Irrigation ditches and canals	Drip irrigation	Livestock
5	Alfalfa	Irrigation ditches and canals	Surface irrigation	Rodents *
6	Alfalfa and walnut trees	Irrigation ditches and canals	Surface irrigation	Rodents *
7	Mixed vegetable–vineyard	Irrigation ditches and canals	Surface irrigation	Rodents *
8	Walnut trees	Water storage ponds and well water	Drip irrigation	Rodents *

* Previous evidence of rodents or pathogenic *Leptospira* infection in rodents corresponds to information reported in previously published studies conducted at the same sites. These data were included as contextual ecological information, were not obtained during the environmental sampling conducted in the present study, and were not included as explanatory variables in the statistical analyses.

**Table 2:** Physicochemical characteristics of water and soil samples collected from agricultural ecosystems of central Chile for the environmental detection of pathogenic *Leptospira*, stratified by sampling season and PCR result. Values are presented as median [Q1–Q3]. Seasonal differences were evaluated using the Kruskal–Wallis test, followed by post hoc pairwise comparisons when appropriate. Differences between PCR-positive and PCR-negative samples were evaluated using the Wilcoxon rank-sum test. Different superscript letters indicate significant differences within the same variable and matrix. For seasonal comparison, letters compare values among winter, spring and summer within each row. For the PCR-result comparison, letters compare PCR-negative and PCR-positive samples within each row. Values sharing the same superscript letter are not significantly different.

Matrix	Variable (Unit)	By Season (Median, [Q1–Q3])					By PCR Result (Median, [Q1–Q3])			
		Winter	Spring	Summer	KW χ^2^	*p*-Value	Negative	Positive	W	*p*-Value
Water	Temperature (°C)	16.8 ^a^ [14.8–18.3]	20.1 ^b^ [18.7–22.7]	23.7 ^c^ [22.7–24.2]	115.27	<0.001	20.8 ^a^ [18.7–23.4]	23.5 ^b^ [21–24.6]	3410	<0.001
	pH	8.1 [7.9–8.2]	8.0 [7.6–8.2]	8.0 [7.8–8.2]	3.67	0.16	8 [7.7–8.2]	8 [7.7–8.2]	5336	0.72
	Conductivity (µS/cm)	2180 ^a^ [1901–2415]	1728 ^b^ [1613–2363]	1494 ^c^ [1211–1665]	66.31	<0.001	1703.5 [1547–2170]	1652 [1409–1835]	5995	0.06
	Turbidity NTU	16.0 ^a^ [7.4–28.4]	27.2 ^b^ [13.3–82.7]	41.3 ^c^ [18.9–266]	15.96	<0.001	26.4 ^a^ [11.3–88.2]	42.6 ^b^ [22.3–142.1]	3378	0.013
Soil	pH	8.4 ^a^ [8.1–8.6]	7.7 ^b^ [7.1–8.0]	7.5 ^c^ [7.0–7.7]	96.26	<0.001	7.7 [7.2–8]	7.6 [7–7.9]	15,773	0.14
	Moisture (%)	36.8 ^a^ [27.2–40.1]	25.3 ^b^ [20.6–31.8]	24.4 ^b^ [19.8–29.4]	30.48	<0.001	24.7 [19.7–34.1]	26.6 [22–34.4]	11,646	0.13

**Table 3:** Results of the binomial GLMM evaluating factors associated with PCR positivity for pathogenic *Leptospira* DNA in environmental samples collected from agricultural ecosystems of central Chile. The sampling site was included as a random intercept. Winter and water were used as reference categories.

Predictor	Comparison	Estimate	Standard Error	Odds Ratio	95% CI	*p*-Value
Season	Spring vs. winter	0.317	0.314	1.37	0.74–2.54	0.31
Season	Summer vs. winter	1.177	0.315	3.25	1.75–6.02	<0.001
Sample Type	Soil vs. water	0.758	0.200	2.13	1.44–3.15	<0.001

## Data Availability

The original contributions presented in this study are included in the article. Further inquiries can be directed to the corresponding author.

## Abbreviations

The following abbreviations are used in this manuscript:

KW	Kruskal–Wallis
Q1	First quartile
Q3	Third quartile
CI	Confidence interval

## Appendix A

**Table A1:** Distribution of PCR-positive samples for pathogenic *Leptospira* by sampling site, environmental matrix, and season. Values are shown as positive samples/total samples (n/N) (%).

Site	Positivity, n/N (%)	By Sample Type n/N (%)		By Season n/N (%)		
		Water	Soil	Winter	Spring	Summer
1	28/69 (40.6)	4/31 (12.9)	24/38 (63.2)	6/19 (31.6)	7/29 (24.1)	15/21 (71.4)
2	13/55 (23.6)	9/28 (32.1)	4/27 (14.8)	2/11 (18.2)	3/24 (12.5)	8/20 (40.0)
3	15/106 (14.2)	2/35 (5.7)	13/71 (18.3)	2/14 (14.3)	9/62 (14.5)	4/30 (13.3)
4	13/30 (43.3)	7/12 (58.3)	6/18 (33.3)	0/5 (0.0)	5/13 (38.5)	8/12 (66.7)
5	21/94 (22.3)	5/39 (12.8)	16/55 (29.1)	2/9 (22.2)	12/59 (20.3)	7/26 (26.9)
6	22/61 (36.1)	5/26 (19.2)	17/35 (48.6)	4/12 (33.3)	12/27 (44.4)	6/22 (27.3)
7	51/145 (35.2)	20/65 (30.8)	31/80 (38.8)	1/9 (11.1)	16/67 (23.9)	34/69 (49.3)
8	13/45 (28.9)	1/12 (8.3)	12/33 (36.4)	0/9 (0.0)	4/18 (22.2)	9/18 (50.0)
TOTAL	176/605 (29.1)	53/248 (21.4)	123/357 (34.5)	17/88 (19.3)	68/299 (22.7)	91/218 (41.7)
